# Utilization of maternal health care services among pastoralist communities in Marsabit County, Kenya: a cross-sectional survey

**DOI:** 10.1186/s12978-024-01865-3

**Published:** 2024-09-02

**Authors:** Dahabo Adi Galgalo, Peter Mokaya, Shalini Chauhan, Evans Kasmai Kiptulon, Girma Alemu Wami, Ákos Várnagy, Viktória Prémusz

**Affiliations:** 1 Doctoral School of Health Science, Faculty of Health Science, University of Pécs, Vörösmarty u. 4, Pecs, 7621 Hungary; 2 Ministry of Health, Marsabit County, Moyale Sub-County, PO Box 57 (60 700) Kenya; 3 National Laboratory on Human Reproduction, University of Pécs, Pecs, 7622 Hungary; 4 Department of Obstetrics and Gynecology, Medical School, University of Pécs, Pecs, 7624 Hungary; 5 HUN-REN-PTE Human Reproduction Research Group, Pecs, 7624 Hungary; 6 Institute of Physiotherapy and Sport Sciences, Faculty of Health Sciences, University of Pécs, Pecs, 7621 Hungary

**Keywords:** Maternal healthcare, Antenatal care, Health facility delivery, Postpartum care, Pastoralist women, Moyale Sub-County, Kenya

## Abstract

**Background:**

Improving maternal healthcare services is crucial to achieving the Sustainable Development Goal (SDG-3), which aims to reduce maternal mortality and morbidity. There is a consensus among different researchers that proper utilization of maternal healthcare services can improve the reproductive health of women, and this can be achieved by providing Antenatal Care (ANC) during pregnancy, Health Facility Delivery (HFD), and Postnatal Care (PNC) to all pregnant women. The main aim of this study was to investigate the utilization and factors associated with maternal and child healthcare services among women of reproductive age in the pastoralist communities in Kenya.

**Methods:**

A cross-sectional survey was conducted among 180 pastoralist women who gave birth in the past two years across ten mobile villages in Marsabit County between 2nd January and 29th February 2019. Three key outcomes were analyzed, whether they attended ANC 4+ visits, delivered at HF, and received PNC. Pearson χ^2^ test and multivariate logistic regression analysis were conducted by IBM SPSS27.0 following Strengthening the Reporting of Observational Studies in Epidemiology (STROBE) guidelines. The significance level was set at p < 0.05.

**Results:**

Of the 180 eligible pastoralist women (mean age 27.44 ± 5.13 years), 92.2% were illiterate, 93.9% were married, 33.3% were in polygamy, and 14.4% had mobile phones. The median commuting distance was 15.00 (10–74) km, 41.7% attended ANC 4+, 33.3% HFD, and 42.8% PNC. Those women residing close (≤ 15 km) to a health facility had a threefold higher ANC 4+ (OR 3.10, 95% CI 1.47–6.53), 2.8-fold higher HFD (OR 2.80, 95% CI 1.34–5.84), and 2.5-fold higher PNC (OR 2.49, 95% CI 1.19–5.22) probability. The likelihood was 30-fold higher for ANC 4+ (OR 29.88, 95% CI 6.68–133.62), 2.5-fold higher for HFD (OR 2.56, 95% CI 0.99–6.63), and 60-fold higher for PNC (OR 60.46, 95% CI 10.43–350.55) in women with mobile phones. A monogamous marriage meant a fivefold higher ANC 4+ (OR 5.17, 95% CI 1.88–14.23), 1.6-fold higher HFD (OR 1.67, 95% CI 0.77–3.62), and a sevenfold higher PNC (OR 7.05, 95% CI 2.35–21.19) likelihood. Hosmer Lemeshow test indicated a good-fitting model for ANC 4+, HFD, and PNC (p = 0.790, p = 0.441, p = 0.937, respectively).

**Conclusion:**

In conclusion, the utilization of three essential maternal health services is low. Geographic proximity, monogamous marriage, and possession of mobile phones were significant predictors. Therefore, it is recommended that stakeholders take the initiative to bring this service closer to the pastoralist community by providing mobile health outreach and health education.

## Plain Language Summary

Attending maternal healthcare clinics is essential to reduce maternal deaths and infections. This can be achieved by receiving antenatal care, delivering at health facilities, and checkups after delivery. We investigated the utilization and factors associated with maternal and child healthcare services among pastoralist women of reproductive age who have given birth in the past two years. Of one hundred and eighty women who participated, most of them were illiterate; the majority were married, of which almost a quarter were in polygamous marriages. This population's uptake of antenatal care, delivery in health facilities, and checkups after delivery is low. This means, that walking distance to the health facility was more than 15 km, almost half of women attended antenatal care and received checkups after delivery but only thirty-three percent delivered at a health facility. Geographic proximity, monogamy, and possession of mobile phones for communication were significant in determining the usage of maternal health care. Living close to a health facility means almost three times more antenatal care, two times more health facility delivery, and checkups after delivery. Women with mobile phones showed twenty-seven more times chances to attend antenatal care, more than four times chances to deliver in a health facility and sixty times more chances of having checkups after delivery. Monogamous marriage showed five times higher odds to attend ANC 4+ visits, and seven times having checkups after delivery. Therefore, it is recommended that stakeholders take the initiative to bring this service closer to the pastoralist community.

## Background

Globally, in the year 2020, nearly 800 women die every day from preventable pregnancy-related causes, with a Maternal Mortality Rate (MMR) of 223 for every 100,000 live births. The majority of this happens in Africa, and Sub-Saharan Africa contributes to over 90% of all maternal deaths [1]. The Sustainable Development Goals (SDGs) of the United Nations (UN), target 3.1, aims to reduce the MMR to less than 70 maternal deaths per 100,000 live births by 2030 in some countries across the globe [2]. Additionally, no country should have twice the maternal mortality rate as the global average [3]. To achieve a global MMR of less than 70 per 100,000 live births by 2030, a year reduction rate of 11.6% is necessary. This rate has not been achieved at the national level in Kenya [4]. Maternal mortality rates in Kenya have decreased from 362/100,000 live births in 2014 to 355/10,000 live births in 2022; this is high when compared to global incidence rates [5]. According to a World Bank Report, MMR incidence in Kenya is higher than the estimated global MMR; this is a worrying indicator in maternal and newborn care [1].

The major complications during pregnancy that contribute to the deaths of pregnant women are haemorrhage, infection, high blood pressure, complications of unsafe abortion, and obstructed labour [6]. Proper Antenatal Care (ANC) during pregnancy, Health Facility Delivery (HFD), and Postnatal Care (PNC) are essential for the health of the mother and baby; if this care can be given to all expectant women, maternal deaths can be prevented, and improves the reproductive health of women [7, 8]. The health status of mothers and children is a crucial indicator in the country; therefore, curbing preventable maternal mortality is critical to achieving SDGs [9].

Marsabit County is the largest county in Kenya, which covers an area of 70,961 km^2^ and is approximately 12% of the territory of Kenya [10]. It borders the republic of Ethiopia to the North, Wajir County to the East, and Isiolo and Samburu Counties to the southwest and southeast, respectively. According to the 2019 Kenya Population and Housing Census, the total population is 459,785 [11]. The county is predominantly inhabited by pastoralist communities, constituting 80% of the population with nomadic pastoralists’ lifestyle centered around their livestock. Marsabit County has a maternal mortality ratio of 811/100,000 live births compared to the national estimate of 355/100,000, as per the 2019 Kenya Population and Housing Census [11]. According to the 2022 Kenya Demographic Health Survey (KDHS) 2022, 66% of Kenyan women had ANC 4+ visits, 82% were delivered in a health facility, and 78% had PNC, of which Marsabit County had 67% ANC 4+ coverage, 59.3% health facility deliveries, and 41% PNC [5].

ANC is an essential maternal healthcare service provided by skilled healthcare professionals that includes taking a gynecological medical history to identify any risk associated with pregnancy, physical examination, laboratory screening, and health education, to ensure a healthy pregnancy and safe childbirth [12]. Adequate ANC visit (i.e., more than four visits) was significantly associated with a reduction of postpartum haemorrhage, early neonatal death, and low birth weight as per a cohort study done in Ethiopia [13].

HFD services are one of the ways of decreasing maternal mortality because the majority of maternal deaths occur during the time of delivery [14]. This death is caused by manageable complications during labor [15] social-demographic factors associated with the quality of intrapartum care [16] and indirect causes [17]. A high percentage of deaths during childbirth can be reduced if women receive skilled birth attendants by qualified health workers in a health facility with adequate resources during childbirth. This approach minimizes the death of pregnant women by early detection of complications [18].

PNC is a vital service healthcare workers provide to women and their newborns immediately following delivery. It extends up to 6 months postdelivery, as is the current practice in Kenya [9]. These health services play an essential role in mothers' and newborns' well-being [19]. Globally, an estimated 60% of annual maternal deaths occur within 48 h after delivery, two-thirds arise within the first-week post-delivery, and over 85% occur within 2 weeks of childbirth [12]. Currently, rates of skilled care post-delivery are lower compared to those before and during delivery. However, the provision of high-quality PNC services can prevent illness and death during this period [20].

Nurses and midwives play important roles by supporting pregnant women throughout the pregnancy journey. Nurses assist with screenings, risk factor assessments, education, and counseling during ante/prenatal care, while midwives care for women during labor, monitoring both the mother and fetus by providing prenatal care, which includes regular check-ups, monitoring fetal growth, and assessing maternal health. Midwives also assess labor progress, manage complications, assist with pain management, and perform episiotomies if needed [21].

Preventive measures and health system intervention to improve the utilization and quality of antenatal care is one of the World Health Organization (WHO) recommendations for positive pregnancy outcomes [22]. Educating pregnant women is essential for promoting maternal and fetal health; this includes; health education and counseling, clinical assessments (fetal assessment blood tests, tetanus vaccination), preventive measures (Malarial and HIV prevention, nutrition, physical activity), and health system interventions (regular ANC contacts, quality improvement) [23].

Giving high-quality maternal healthcare services is crucial for the overall well-being of pregnant women during childbirth and in the postpartum phase. Research has consistently demonstrated that adequate maternal healthcare services, including regular ANC visits, HFD, and PNC, can significantly decrease maternal mortality and morbidity rates [24–27]. Despite progress made globally and by the government of Kenya to decrease maternal fatalities and enhance maternal healthcare services, challenges remain with remote communities, such as pastoralist communities. The pastoralist communities lack access to healthcare services because of the nature of their movement from one place to another in search of pasture and water, and usually static health services do not often address their healthcare needs. They also experience long-standing marginalization, poverty, and vulnerability [28].

World Health Organization (WHO) recommended that the distance from every inhabitant to the nearest health care service should not exceed 5 km [29]. Most pastoralist communities travel more than 10 km to seek healthcare services, limiting access to healthcare services [30]. However, there remains a lack of comprehensive research on maternal healthcare service utilization among pastoralist communities in the upper counties of Kenya. Therefore, this study aims to assess the uptake of ANC, HFD, and PNC and factors associated with its use among women of reproductive age (15–49) in ten mobile/pastoralist communities in the Moyale sub-county of Marsabit County, Kenya. It’s essential to understand this because the findings of this research will inform the policy on appropriate strategies for providing maternal health services for pastoralist women.

## Methods

### Sampling

The data for this research was collected as part of the 2019 baseline survey conducted for the Integrated Maternal Mobile Health (IMMH) Clinic. The IMMH Clinic was a research project supported through Grand Challenge Africa (GCA) as a seed grant to bring an innovative idea to decrease maternal and newborn deaths among pastoralist mothers through provisional of antenatal, and postnatal care in Moyale Sub-County of Marsabit County in Kenya. A probability sampling technique was applied, whereby mobile communities that lived a nomadic lifestyle and moved with their families and animals in search of pasture and water were grouped into a two clusters, which include; those with ongoing health intervention and those without intervention. Ten mobile/pastoralist villages from the selected cluster without intervention were selected randomly from thirty-eight mobile villages using a computer-generated method by an independent researcher [31]. These villages include Chirach, El-Raya, Er-Wede, Funandimo, Laqi, Mansile Water Point, Qalaliwe, Qilta, Tesso, and Yaballo Godha. A purposive sampling technique was applied to enroll the participants from the randomly selected mobile village until the sample size was reached [32].

The survey used a cross-sectional study, eligible participant was selected through purposive sampling techniques, and study participants were mobile pastoral pregnant women in their first trimester of pregnancy. Study site visits were conducted to interact with key community leaders and stakeholders to sensitize them and educate women representatives of the community for the research. All women of reproductive age were identified by community women leaders (matriarchs), traditional birth attendants, community health workers, community health volunteers, other pregnant women, and husbands of pregnant women. Community health workers listed all identified women of reproductive age during health service outreach visits.

**Fig. 1:**
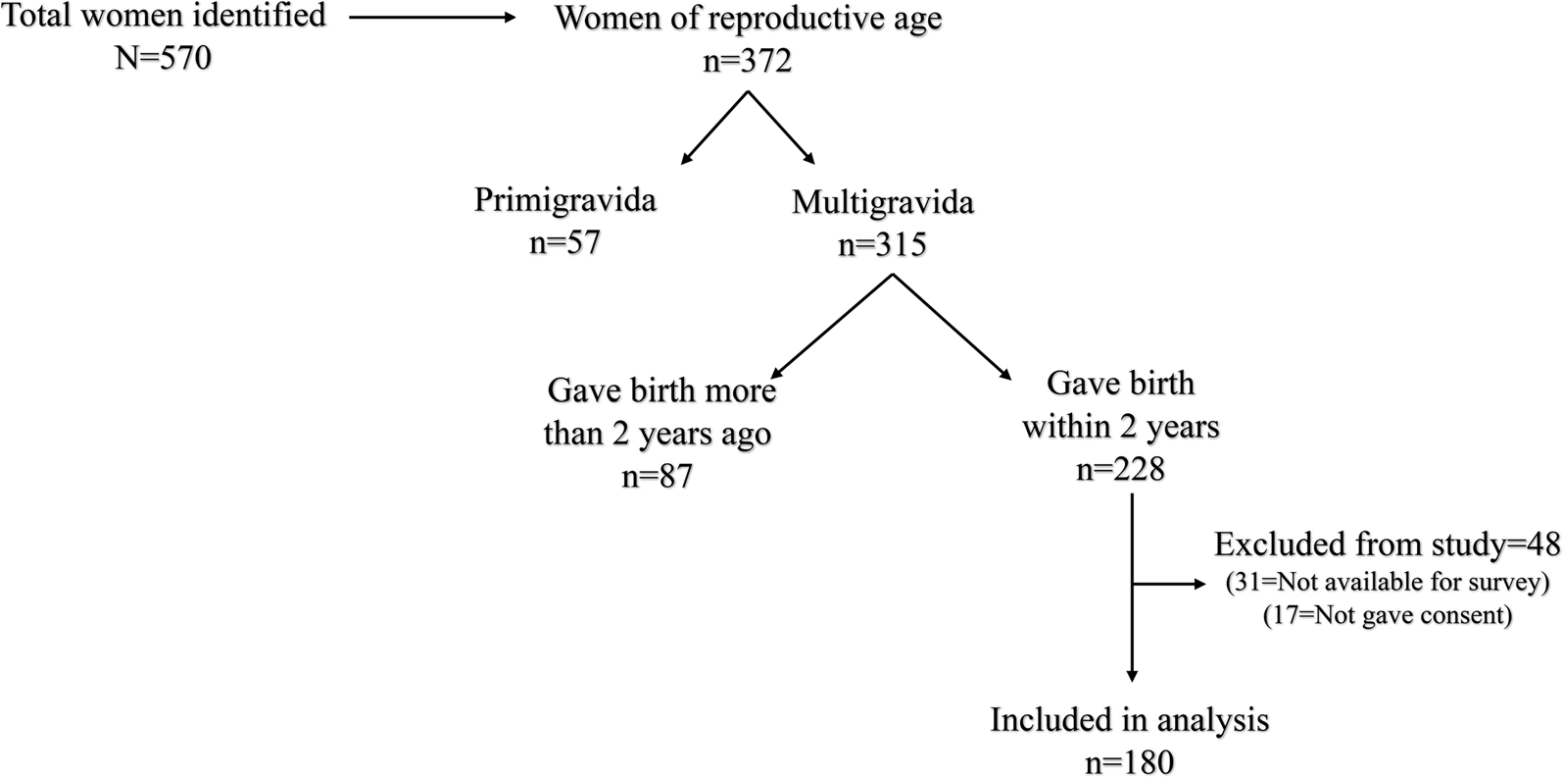
Prisma diagram showing a sampling procedure of women to be included in the study population

At the first stage, the total number of women identified within randomly selected mobile villages was 570; of these, 372 were women of reproductive age, of whom 315 were multigravida and the remaining 57 were primigravida. All multigravida women were asked willingly to attend mobile outreach organized by the IMMH team, and women who voluntarily attended and gave consent were screened using a standard questionnaire to determine eligibility. The inclusion criteria were women of reproductive age (15–49 years), nomadic/pastoralism, multigravida, who gave birth less than 2 years ago, and verbal consent. Of the 315 multigravida women, 228 had given birth less than 2 years ago. During the screening, 48 participants were excluded from the study; of those excluded, 31 were not available for the interview, and 17 did not give consent. A total of one hundred and eighty women who met the inclusion criteria and agreed to participate were included in the survey and subsequent analysis (Fig. 1). Data collection took place between 2nd of January to the 29th of February 2019.

### Data collection

The study utilized a structured questionnaire developed from existing literature and published studies written in English and modified to the local language. The questionnaire consists of socio-demographic information, clinical information, and past pregnancy history. The data collection was performed using a standardized questionnaire, with the help of 22 trained healthcare workers consisting of nurses, public health officers, medical laboratory technologists, and clinical officers fluent in Oromo, Kiswahili, and English. The supervisors were two epidemiologists with master’s degrees and five years of experience in field research. All the data collectors underwent a comprehensive three-day training program, which included guidance on using data collection tools, interviewing techniques, research ethics, and using tablets for data collection and paper-based questionnaires as a backup in case of power shortage or network issues. The training was concluded by visiting the nearby village for practical exercises on using instruments on a similar population, which was not part of the baseline survey. Every mobile village was assigned a team of two data collectors supervised by a team leader to ensure that all the data collected was clean with no missing variables and was uploaded at the end of the day.

### Data quality control measures

Data quality was ensured through validation checks, random spot checks, and daily supervision, and any issues identified during the supervision were thoroughly discussed the following morning before the commencement of the day's data collection activities. All consented participants were told about the importance and significance of the baseline survey; if any participants selected for the study refused to participate, the next eligible respondent was interviewed. Checking the completeness of questionnaires was performed daily by the research team lead, and in case of incompleteness of questionnaires noted in the process, they were referred to the data collectors, who revisited the respondents to complete the questionnaires.

Different variables were chosen based on thorough relevant literature sources [9, 27, 33, 34]. Three outcome measures of maternal health care service use were evaluated, i.e., the number of ANC visits, the place of childbirth i.e., HFD, and PNC whether given or not. As per WHO recommendation, the standard for the number of recommended ANC visits is more than four; therefore, women who visited at least four visits were assigned a score of 1, while those who visited none or fewer than four visits received a score of 0. The assessment of childbirth was done by asking a woman about the location of the last pregnancy childbirth; those women who delivered in a formal private or public medical facility with the assistance of a health professional were categorized into health facility delivery and coded 1, and 0, if delivery takes place at home without the assistance of any health professional. Postpartum care was assessed by asking each woman whether a healthcare worker had checked on her health after delivery, irrespective of the place of delivery. Postnatal care was coded as 1 if a woman received any form of postnatal care, and 0, if she did not undergo postnatal care.

Independent variables included the maternal age at the time of the survey, marital status, literacy level, distance to health facilities from the mobile village, and having or not having co-wives. The geographical proximity of the health facilities from the mobile village was measured in kilometers, which was categorized as less or equal to 15 km (< = 15) as 1 and more than or equal to 16 km (> = 16) as 0. The age of the participants was recorded on a continuous scale and then grouped into four categories: 15–19, 20–29, 30–39, and 40–49 years. Marital status was collected as a categorical variable, such as married, divorced, or widowed, later coded as married as 1, divorced, and window was merged as unmarried and coded 0. Maternal literacy level was dichotomized as literate 1 and illiterate 0. Those women whose husbands were married to more than one wife at the same time were categorized as polygamous and coded as 1, and those whose husbands were married to only one wife were recorded as monogamous and coded as 0. Lastly, those with mobile phones for communication with healthcare workers were categorized as 1, and those without mobile phones were recorded as 0.

### Data analysis

Our study was conducted and data were reported by the Strengthening the Reporting of Observational Studies in Epidemiology (STROBE) guidelines [35]. The data analysis process involved three steps. First, descriptive analysis was conducted, which included calculating the mean ± standard deviation (SD) for continuous variables and the median (interquartile range [IQR]) along with frequencies for categorical variables. Second, bivariate analysis was performed using the chi-square test to identify associations between individual factors and various outcome measures and to select relevant variables for the multivariate analysis.

Finally, multivariate logistic regression was carried out, and in the model, the dependent variable was binary coded; this approach explored the impact of multiple independent variables on the binary outcome, which provided a clear understanding of the dataset. The predictive factors were chosen by fitting a logistic regression model using a forward selection method procedure (p < 0.05), and independent variables that show significance in bivariate analysis were introduced for pre-testing. All significantly contributing variables were introduced in the final prediction model and analyzed according to their categories in a multivariate logistic regression analysis with the entered methods.

The model testing was done using, the Omnibus Test of model Co-efficient, Nagelkerke R Square, classification table, Hosmer–Lemeshow test, and Area under ROC curve (AUC).

Omnibus test of model co-efficient is used to check if the model is statistically significant, it uses the chi-square tests to see if there is a significant difference between the log Likelihoods of the baseline model and the new model. The Omnibus test of model co-efficient shows if the p-value is < 0.05, the model is statistically significant and adequately describes the data. As per Hosmer–Lemeshow goodness-of-fit, the model can be considered a well-calibrated and good-fitting model when its p-value is more than > 0.05; this implies that the model estimates fit the data at an acceptable level. To measure the ability of the discriminating power of a chosen model, the area under the ROC curve > 0.7 (with 95% confidence limits) was considered an acceptable fit. A classification table summarized a comparison between the observed and predicted classifications, considered a good model fit with 70% [36].

After cleaning and coding the dataset, analysis was conducted using IBM SPSS version 28.0 (SPSS Inc., Chicago, IL, USA). The significance level was set at p < 0.05.

## Results

### Sample characteristics

A total of 180 pastoralist women participated in the baseline survey. Their average age was 27.44 ± 5.13 years, and the majority were in the age group of 20–29, comprising 108 people (60.0%). Of all the participants, 169 (93.9%) were married, 33.3% practiced polygamous and 166 (92.2%) were illiterate. Only 26 (14.4%) had a mobile phone, and most of them, 51 (28.3%), came from Yaballo Godha village. The median distance traveled to reach the health facility was 15.00 km with Inter Quartile Ranges (IQR) of (10.00 and 74.00). Most of the participants, 60 (33.3%), visit the Mansile dispensary. Regarding the use of contraceptives, 146 (81.1%) reported that they had never used contraceptives as a form of family planning, and 127 (70.6%) had never received advice on their use or did not know where to obtain them. On assisted birth, most of them 106 (58.9%) were assisted by Traditional Birth Attendants (TBA), 45 (25%) by Health Care Workers (HCW), and 29 (16.1%) by both TBA and HCW. Approximately 75 individuals (41.7%) have visited ANC 4+; 60 (33.3%) had HFD, and only 77 (42.8%) received PNC (Table 1).

**Table 1:** Socio-demographic information of study participants in Moyale Sub-County, of Marsabit County (N = 180)

Characteristics	Frequency (%)	Characteristics	Frequency (%)
Maternal age, years		Nearest health facility	
Mean (SD)	27.44 (5.13)	Kate Dispensary	3 (1.7)
15–19	8 (4.4)	Mansile Dispensary	60 (33.3)
20–29	108 (60)	Odda Dispensary	52 (28.9)
30–39	60 (33.3)	Somare Dispensary	14 (7.8)
40–49	4 (2.2)	Yaballo Dispensary	51 (28.3)
Marital status		ANC attendance	
Married	169 (93.9)	< 4 Visit	105 (58.3)
Widowed	5 (2.8)	> 4 Visit	75 (41.7)
Divorced	6 (3.3)	Location of birth	
Polygamous family		Health facility delivery	60 (33.3)
Yes	60 (33.3)	Home delivery	120 (66.7)
No	120 (66.7)	Assisted birth	
Have mobile phone		Health care worker	45 (25)
Yes	26 (14.4)	HCW/TBA	29 (16.1)
No	154 (85.6)	TBA	106 (58.9)
Village of residence		Postnatal care	
Chirach	18 (10)	Yes	77 (42.8)
El-Raya	7 (3.9)	No	103 (57.2)
Er-Wede	4 (2.2)	Use of contraceptive	
Funandimo	47 (26.1)	Yes	34 (18.9)
Laqi	3 (1.7)	Not	146 (81.1)
Mansile Water Point	9 (5)	Advised on birth control	
Qalaliwe	34 (18.9)	Yes	53 (29.4)
Qilta	1 (0.6)	No	127 (70.6)
Tesso	6 (3.3)	Know where to get contraceptive	
Yaballo Godha	51 (28.3)	Yes	53 (29.4)
Distance to HF (km)		No	127 (70.6)
Median (IQR)	15 (10–74)	Literacy level	
≤ 15	109 (60.6)	Literate	14 (7.8)
≥ 16	71 (39.4)	Illiterate	166 (92.2)

Freq: Frequency; ANC: Antenatal care; HCW: health care worker; TBA: traditional birth attendance; H/F: health facility

### Results of bivariate analysis of maternal health care service

According to the bivariate analysis (Table 2)*,* some of the independent variables show a significant association with the dependent ANC 4+ visit, health facility delivery, and postnatal care.

**Table 2:** Bivariate analysis of maternal healthcare services utilization of pastoralist women according to sample characteristic (N = 180)

Background characteristic	Total (N = 180)	ANC (4+)		Health facility delivery (n = 60)		Postnatal care (n = 77)	
		(n = 75)					
		n (%)	p-value	n (%)	p-value	n (%)	p-value
Women age			0.108		0.187		0.183
15–19	8	1 (12.5)		0 (0)		1 (12.5)	
20–29	108	42 (38.9)		36 (33.3)		45 (41.7)	
30–39	60	31 (51.7)		22 (36.7)		30 (50)	
> 40	4	1 (25.0)		2 (50)		1 (25)	
Marital status			0.793		0.439		0.661
Married	169	70 (41.4)		55 (32.5)		72 (42.6)	
Not Married	11	5 (45.5)		5 (45.5)		5 (45.5)	
Polygamous			0.054		0.314		0.033
Yes	60	19 (31.7)		43 (71.7)		19 (31.7)	
No	120	56 (46.7)		17 (14.2)		58 (48.3)	
Mobile phone			0.001		0.016		0.001
Yes	26	23 (88.5)		14 (53.8)		24 (92)	
No	154	52 (33.8)		46 (29.9)		53 (34.4)	
Literate level			0.110		0.115		0.093
Literate	14	3 (21.4)		2 (14.3)		3 (21.4)	
Illiterate	166	72 (43.4)		58 (34.9)		74 (44.6)	
Distance to HF (km)			0.001		0.001		0.001
≤ 15	109	60 (55.0)		47 (43.1)		60 (55.0)	
≥ 16	71	15 (21.1)		13 (18.3)		17 (23.9)	
ANC 4+ visit					0.001		0.001
< 4	105			15 (14.3)		4 (3.8)	
> 4	75			45 (60)		73 (97.3)	
Place of delivery			0.001				0.001
HFD	60	45 (75.0)				48 (80.0)	
Home Delivery	120	30 (25.0)				29 (24.2)	
PNC			0.001		0.001		
Yes	77	73 (94.8)		48 (62.3)			
No	103	4 (3.9)		29 (28.1)			

Pearson χ^2^-p ≤ 0.01; ANC: antenatal care; km: kilometer; PNC: postnatal care; HFD: health facility delivery; HF: health facility

Women in monogamous marriages were significantly more likely to have ANC 4+ visits p = 

0.054, (n = 56; 46.7%), and postnatal care, p = 0.033, (n = 58; 48.3%), compared to women in a polygamous marriage, however, no statistically significant difference was noted in the likelihood of health facility delivery.

Having a mobile phone for communication was significantly predictive of ANC 4+ visits, HFD, and receiving PNC. Women with mobile phones are likely to have ANC 4+ visits p = 0.001, (n = 23, 88.5%), HFD p = 0.020, (n = 14, 53.8%), and PNC p = 0.001, (n = 24, 92%).

The distance between the health facility and the village of residence was significantly associated with ANC 4 + visits, HFD, and PNC, with women living less than 15 km from the health facility being more likely to receive ANC 4 + visits p = 0.001, (n = 60, 55.0%), HFD p = 0.001, (n = 47, 43.1%), and PNC p = 0.001), (n = 60, 55%) than women who leave more than 16 km away from health facilities.

Attending ANC 4 + visits were significantly associated with HFD and receiving PNC, with women who attended ANC 4+ more likely to deliver at a health facility, p = 0.001, (n = 45, 60%) and receive PNC, p = 0.001, (n = 73, 97.3%). Variables such as maternal age, marital status, and literacy level did not indicate any association with ANC 4+ visits, HFD, and PNC.

### Multivariable analysis of maternal health service utilization

The multivariate analysis results are presented in (Table 3). Many variables that demonstrated significant differences in the bivariate analyses shown also significance in the multivariable analysis. Notably, factors such as possession of a mobile phone, monogamous family, and distance to the health facility retained their importance with ANC 4+ visits, HFD, and PNC. After accounting for confounding factors, other variables did not exhibit a statistically significant association.

**Table 3:** Logistic regression of maternal healthcare service utilization of pastoralists women in Moyale sub-county by sociodemographic factors

Dependent variables	Independent variables	B	S.E	Wald	df	Sig	Exp(B)	95% CI for EXP(B)	
								Lower	Upper
ANC 4 + Visit (N = 75)	Polygamous								
	Yes	Ref							
	No	1.643	0.517	10.118	1	0.001	5.171	1.879	14.232
	Distance to HF								
	≤ 15	1.131	0.381	8.834	1	0.003	3.100	1.470	6.536
	≥ 16	Ref							
	Mobile phone								
	No	Ref							
	Yes	3.397	0.764	19.758	1	0.001	29.876	6.680	133.616
	Constant	− 2.672	0.536	24.828	1	0.001	0.069		
Health Facility Delivery (N = 60)	Polygamous								
	Yes	Ref							
	No	0.513	0.394	1.689	1	0.194	1.670	0.771	3.618
	Distance to HF								
	≤ 15	1.030	0.375	7.524	1	0.006	2.800	1.342	5.843
	≥ 16	Ref							
	Mobile phone								
	No	Ref							
	Yes	0.939	0.486	3.736	1	0.053	2.558	0.987	6.630
	Constant	− 1.873	0.416	20.235	1	0.001	0.154		
Postnatal care (N = 77)	Polygamous								
	No	1.953	0.562	12.097	1	0.001	7.049	2.345	21.189
	Yes	Ref							
	Distance to HF								
	≤ 15	0.913	0.378	5.850	1	0.016	2.492	1.189	5.224
	≥ 16	Ref							
	Mobile phone								
	No	Ref							
	Yes	4.102	0.897	20.925	1	≤ 0.001	60.458	10.427	350.546
	Constant	− 2.765	0.573	23.321	1	≤ 0.001	0.063		

ANC: Antenatal care; OR: Odds ratio p < 0.001; CI: 95% confidence interval; HF: Health Facility

Women residing close to a health facility displayed a threefold higher likelihood of attending up to ANC 4+ visits (OR 3.10, 95% CI 1.47–6.53), a 2.8-fold higher likelihood of delivering to health facilities (OR 2.80, 95% CI 1.34–5.84), and a 2.5-fold higher likelihood of having PNC done (OR 2.49, 95% CI 1.19–5.22), compared to those women living more than 15 km away from a health facility.

Additionally, women with a mobile phone displayed a 30-fold higher likelihood of attending up to ANC 4+ visits (OR 29.88, 95% CI 6.68–133.62), threefold higher likelihood of HFD (OR 2.56, 95% CI 0.99–6.63), and a 60-fold higher likelihood of having PNC (OR 60.45, 95% CI 10.43–350.55), compared to those women who do not possess mobile phone.

Finally, women in monogamous marriage displayed a fivefold higher likelihood of attending up to ANC 4+ visits (OR 5.17, 95% CI 1.88–14.23), likelihood of HFD (OR 1.67, 95% CI 0.77–3.62), and a sevenfold higher likelihood of having PNC (OR 7.05, 95% CI 2.35–21.19), compared to those women in polygamous marriage.

Three goodness-of-fit assessment methods, the Hosmer Lemeshow test, Classification table, and Area under the Receiver Operating Characteristic (ROC) curve were applied for all three dependent variables.

The Omnibus tests of model coefficients showed p < 0.001 for ANC 4+, p = 0.001 for HFD, and p < 0.001 for PNC; these results showed all p-values are less than 0.05, which indicates a very good model fit.

Nagelkerke R Square indicated (R^2^N = 0.356) for ANC 4+ visits, (R^2^N = 0.124) for HFD, and (R^2^N = 0.382) for PNC, a moderate influence between the dependent and independent variables. Hosmer Lemeshow goodness-of-fit results show that the p-values for ANC 4+, HFD, and PNC were 0.790, 0.441, and 0.937, respectively. P-values greater than 0.05 indicated a good-fitting model.

ROC results for ANC 4+ visits, HFD, and PNC were 0.782 (0.715–0.849), 0.680 (0.602–0.765), and, 0.787(0.721–0.853) respectively. This means the area under the ROC curve was more than 70.0% correctly classified by the model, which showed more than 70% accuracy for ANC 4+ and PNC but slightly low for HFD. All three methods of model fit assessment indicated good model fit.

## Discussion

Antenatal care, health facility delivery, and postnatal care services are very important for reducing harm to both the mother and newborn child; in case of any complication, intervention can be taken on time. However, delivering at home is common among pastoralist communities in the upper part of Kenya, and the majority of these pregnant women have never received proper antenatal care or postnatal care [37]. The findings of our study reveal that only 41.7% of mothers have received up to a fourth ANC visit, 33% received health facility delivery, and 43% attended postnatal care. The ANC 4 + visit and health facility delivery results are low compared to KDHS 2022, which shows 67% and 59.3% respectively [5]. The proportion of pregnant women from pastoralist counties who received postnatal care during the first two days after a live birth is lowest in Wajir (37%), Marsabit (41%), Garissa (45%), and Mandera (46%), if compared to (72.5%) reported at the national level [5]. The results indicated that the use of antenatal care, utilization of health facilities for delivery, and postnatal care among the pastoralist community in Marsabit County is very low.

The literacy level in Marsabit County is also very low, with almost 68% of residents having no formal education [38]; additionally, Kenny et al. also found that the literacy rate of the nomadic community is very low [39]. Our study confirms the finding that 92% of study participants do not know how to read and write, which could be a contributing factor to low maternal healthcare service utilization. Evidence from the review studies also shows a positive correlation between maternal care services and literacy level, with women's literacy level directly associated with better utilization of health care services. In another study done in Kenya, a lack of education was the key factor preventing women from having health facility deliveries; despite efforts by community health workers to promote awareness about maternal healthcare services, only better-educated or younger women opted for skilled birth attendants during childbirth [37].

A place where women give birth and receive care are crucial in determining the health outcomes of both mother and newborn. The current results also revealed that 120 (66.7%) births occurred at home, with 41.1% of deliveries assisted by trained birth attendants. This finding is similar to the findings of a previous study performed in Kenya showing that almost over fifty percent of all deliveries were conducted under the supervision of unskilled professionals at home [40] and 81% in the Ganda Community from the eastern Indian states of Odisha and Chhattisgarh [41]. Additionally, these findings are consistent with studies done in Africa and developing countries in other continents [42]. One of the studies finds that the majority of home births were attended by nonprofessional personnel [43]. This might be linked to physical distance and transportation constraints that prevent women from accessing healthcare facilities. Some factors that play a significant role in the utilization of healthcare facilities within pastoralist communities include accessibility, the location of nearest hospitals, and means of transport [44].

The WHO recommended that the average distance from every inhabitant to the nearest health care service be at least 5 km. In this study, the majority of the participants walked more than 15 km to seek health services, which may be a factor that prevented them from receiving the required care. Similarly, other studies from Zambia [44] and Nepal [45, 46] found that women who lived further away from health centers providing services were less likely to seek maternal and child health care services. This might be attributed to the unavailability of means of transport, especially in remote areas within pastoralist set-up, and bad road conditions, which make it very difficult for women to reach the nearest health facilities for care. Their cultural beliefs and social norms guide pastoralist women's healthcare-seeking behaviors. The low utilization of these services within pastoralist communities may be attributed to many factors [33, 47, 48], which may affect their health status.

Women in monogamous marriages were significantly more likely to have ANC 4+ visits and PNC compared to women in polygamous marriages. This finding aligns with a previous study conducted in Uganda [49], which indicates, that women should receive support from their husbands to take advantage of antenatal care services. However, in a polygamous relationship, a husband's attention is divided between his wives, which means that he may have less time to attend to each wife’s needs. Therefore, it is necessary to educate husbands about the significance of antenatal care services during pregnancy and encourage them to support their wives in utilizing these services.

Mobile phone use was a significant predictor of ANC +4 visits and PNC, with women who had mobile phones being more likely to have four ANC visits and receive PNC. These findings are consistent with another previous study [50] showing that cell phones for communication can increase the uptake of maternal and child health services. However, many people in this community are regularly on the move in search of pasture and water, making it difficult for healthcare workers to track and provide necessary healthcare services. A lack of mobile phones further complicates this issue.

Finally, women who visited antenatal clinics more than four times were more likely to give birth at health facilities and receive postnatal care than those who had less than four visits. Those women who chose to deliver at health facilities were more likely to receive postnatal care than those who did not deliver at health facilities; this shows a positive correlation between the choice of delivery location and subsequent access to postnatal care. These findings are similar to a previous study in Ethiopia [51], where women who gave birth in a health facility were more likely to be assisted by trained health professionals who gave talks on health education about postnatal care. Missing this service could put the lives of mothers and infants at risk [34]. The positive relationship between health facility delivery and postnatal care may be because these women had the opportunity to receive health education related to postnatal care services during their stay in the health clinics; this could explain why they were more likely to seek out these services [52].

## Limitations

This study focused exclusively on women who gave birth less than two years ago within pastoralist/mobile communities, which approach may lead to recall bias, as women were asked about their children born within the past two years. The results cannot be generalized for all women in Marsabit County because only the mobile pastoralist community who moved with their animals were considered to participate in the study.

## Conclusion and recommendation

This is the first study done in Marsabit County to determine the utilization of maternal and child health care services within pastoralist communities, and our findings showed low coverage of four antenatal attendants, delivering at health facilities and being checked for postpartum after 24 h of delivery among pastoralist women. The findings of this research call for community-led interventions aimed at raising awareness and encouraging early initiation of antenatal care services, focusing on women with low literacy levels. A significant effort should be put into strengthening antenatal visits, anticipating that this will result in increased usage of health facility deliveries and postpartum check-ups.

World Health Organization recommended that the average distance from every inhabitant to the nearest health care services should be at least 5 km (km). In this study, geographic proximity and village of residence are significant in predicting the use of health services for women, with those living farther away being less likely to receive care. To resolve these issues, the Ministry of Health, Marsabit County, stakeholders, and other partners should consider innovative strategies, like the provision of mobile health outreach clinics for easy access to antenatal care, skilled delivery, and postpartum checkups in remote areas with limited access to health facilities.

The results of the current survey may also provide evidence and guidance for policymakers and decision-makers to increase the coverage and quality of critical maternal and child health indicators among pastoralist women.

## Data Availability

Data supporting the study's findings are available from Track and Save a Life (TASAL), a Community-Based Organization (CBO). Data are available upon reasonable request from the authors and with the permission of the Chief Executive Officer (CEO) of TASAL.

## Abbreviations

ANC: Antenatal care
CI: Confidence interval
HMT: Health management team
IQR: Interquartile range
IMMH: Integrated Maternal Mobile Health
KDHS: Kenya Demographic Health Survey
MCH: Maternal and child healthcare
MMR: Maternal mortality rate
OR: Odds ratio
PNC: Postnatal care
SBA: Skilled birth attendant
SD: Standard deviation
SDG: Sustainable Development Goal
TBA: Traditional birth attendant
WHO: World Health Organization
